# Swyer–James–Macleod syndrome mimicking pulmonary embolism in adults: a case report

**DOI:** 10.1186/s13256-024-04680-3

**Published:** 2024-08-08

**Authors:** Girma Deshimo Lema, Ermiyas Endewunet Melaku, Haile Abebe Tekle, Getachew Bizuneh Aydagnuhm, Enguday Demeke Gebeyaw

**Affiliations:** 1 Department of Internal Medicine, Asrat Woldeyes Health Science Campus, Debre Berhan University, Debre Berhan, Ethiopia; 2 Division of Radiology, Department of Internal Medicine, Asrat Woldeyes Health Science Campus, Debre Berhan University, Debre Berhan, Ethiopia; 3 School of Public Health, Asrat Woldeyes Health Science Campus, Debre Berhan University, Debre Berhan, Ethiopia

**Keywords:** Swyer–James–MacLeod syndrome, Bronchiectasis, Lung hyperlucency, Pulmonary embolism, Adult onset

## Abstract

**Background:**

Swyer–James–MacLeod syndrome (SJMS) is a rare lung condition characterized by a unilateral lung hyperlucency and reduction in the pulmonary vasculature, with or without the presence of bronchiectasis. In the 1950s, Swyer, James, and Macleod simultaneously characterized the syndrome for the first time. It is typically diagnosed in childhood. Adult-onset cases are extremely rare, with little literature available on its clinical presentation and diagnostic challenges. Swyer–James–MacLeod syndrome can mimic other lung disorders, resulting in misdiagnosis and improper treatment.

**Case presentation:**

A 49- year-old woman from Debre Berhan, Ethiopia, presented to the emergency department of Hakim Gizaw Teaching Hospital with symptoms and radiographic findings mimicking acute pulmonary embolism. On the basis of the clinical presentation and radiographic findings, the patient was first treated as a probable case of pulmonary embolism. Anticoagulant therapy and oxygen support were initiated. Nevertheless, additional testing using a chest computed tomography angiography revealed left lung hyperlucency, decreased vascularity, bronchiectasis, and a negative result for pulmonary embolism. As a result, Swyer–James–MacLeod syndrome was diagnosed.

**Conclusion:**

The symptoms of Swyer–James–MacLeod syndrome can be mistaken for pulmonary embolism, which could lead to ineffective treatment and needless expenses. In individuals presenting with symptoms suggestive of pulmonary embolism, this case emphasizes the significance of considering Swyer–James–MacLeod syndrome as a differential diagnosis, especially in the absence of established risk factors for pulmonary embolism.

## Introduction

Swyer–James–MacLeod syndrome, also known as Swyer–James syndrome or hyperlucent lung syndrome, is an uncommon syndrome of unilateral functional hypoplasia of the pulmonary vasculature and lung hyperlucency, with or without associated bronchiectasis [1, 2]. SJMS is named after the physicians who initially described the condition. Swyer and James, Canadian physicians, first reported the syndrome in 1953. Macleod, an English pulmonologist, followed up with additional information in 1954. As a result, the condition became known as Swyer–James–Macleod syndrome. This lung condition is characterized by the radiographic hyperlucent appearance of a single pulmonary lobe or the entire lung [3–5].

SJMS has a reported prevalence of 0.01%, with children accounting for the vast majority of cases and adults accounting for only a handful [6]. SJMS is a rare consequence of recurrent pulmonary infections in children, most commonly bronchiolitis obliterans. It has been observed that SJMS occurs in roughly 4% of patients with bronchiolitis obliterans. Several infectious agents have been implicated. The left lung is preferentially involved in most cases for unknown reasons [7, 8].

While some Swyer–James–MacLeod syndrome patients experience recurring lung infections, the majority of these patients are asymptomatic. Some patients are diagnosed in childhood, and others incidentally during adulthood when undergoing investigations for a different reason [9]. This rare disorder is difficult to diagnose because its symptoms are non-specific and can mimic other respiratory conditions. Due to similar clinical manifestations, it is frequently mistaken as chronic obstructive pulmonary disease, asthma, pneumothorax, or pulmonary embolism [10].

The scarcity of research and case studies on adult-onset SJMS emphasizes the need for greater research and case reports to better understand the presentation, diagnosis, and management of this unusual disorder. Increased awareness among healthcare providers may result in earlier detection and management of adult-onset SJMS. Adult-onset SJMS is extremely unusual, with little literature on its clinical presentation and diagnostic challenges.

We present a case of an adult patient with SJMS who came with symptoms and radiographic evidence suggestive of pulmonary embolism.

## Case presentation

A 49- year-old woman from Debre Berhan, Ethiopia, presented to Hakim Gizaw General Hospital emergency department with complaints of a productive cough with blood-tinged sputum, pleurtic chest pain, and dyspnea of 2 days duration. Four months back she had been treated for a lung infection at a nearby health facility. Otherwise, she had no history of diagnosed tuberculosis or bronchial asthma. She had no smoking history. The patient had no noteworthy family history and no prior history of recurring chest infections as a child. She had no previous thromboembolic events.

Upon physical examination, she was in distress. She had a heart rate of 104 beats per minute, a respiratory rate of 30 breaths per minute, an oxygen saturation of 86% while breathing room air, a temperature of 36.8 °C, and a blood pressure of 130/85 mmHg. She was obese, with a body mass index of 33.5 kg/m^2^. During chest examination, crackles were detected in the left posterior upper lung field. There were no precordial findings or peripheral edema.

**Fig. 1:**
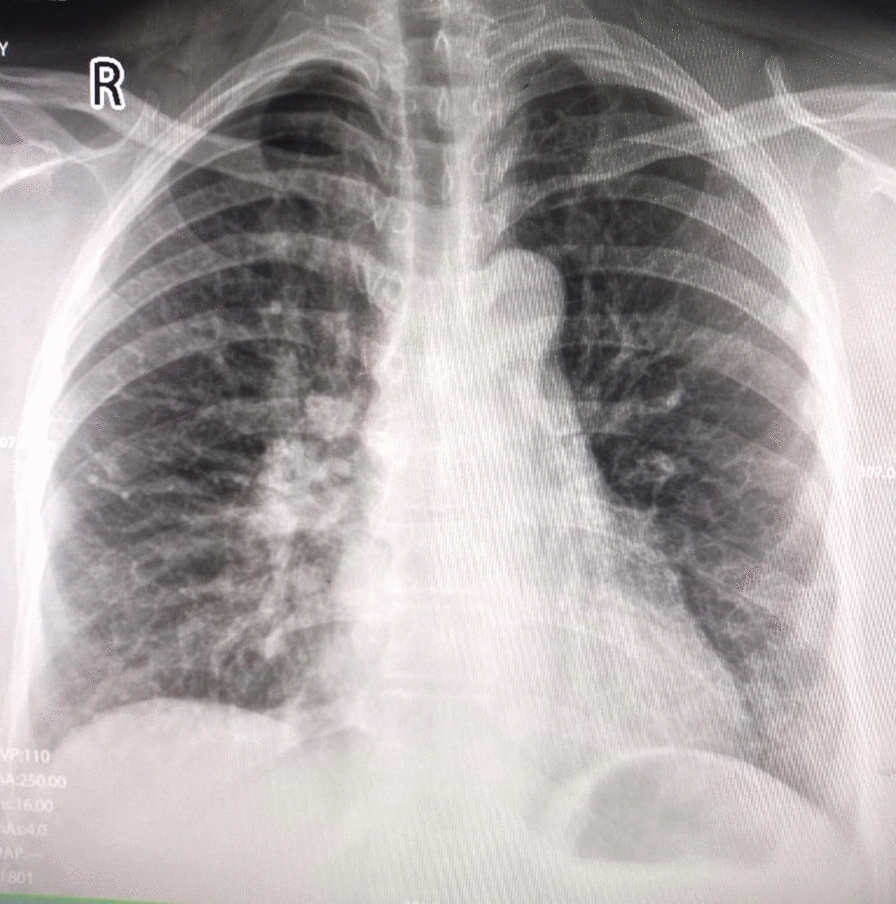
Posteroanterior view of chest X-ray showing left upper lobe hyperlucency and ipsilateral small hila

**Fig. 2:**
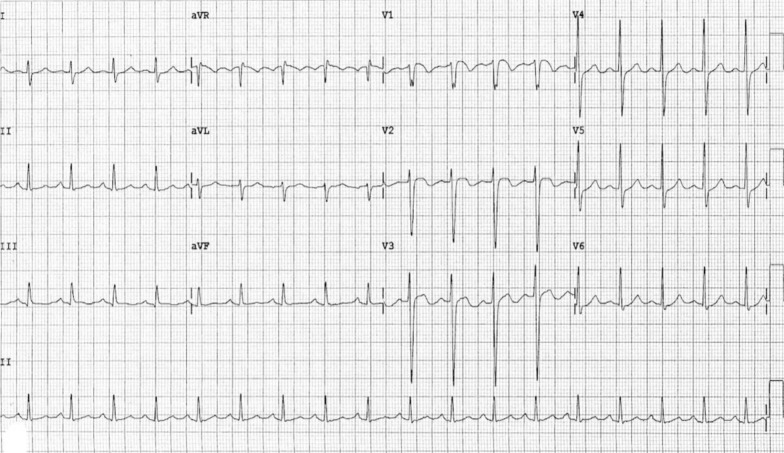
The electrocardiogram showing sinus tachycardia and T wave inversions in leads V1–V3

Upon laboratory investigation, complete blood count showed mild leucocytosis with a total white count of 12 × 10^3^/mm^3^ and absolute neutrophil count of 7.8 × 10^3^/mm^3^. Blood sugar and other biochemical tests were within normal limits. An X-ray of the chest revealed hyperlucency in the left upper lobe, which was initially described as oligemia (Fig. 1). Her electrocardiogram (ECG) showed sinus tachycardia and T wave inversions in leads V1–V3 (Fig. 2). The Wells probability score was moderate.

Considering the clinical presentation and radiographic findings, the patient was initially treated as a suspected case of submassive pulmonary embolism (PE). Oxygen support and anticoagulation therapy were initiated. Following this, the patient was admitted to the medical ward, and additional testing was planned.

**Fig. 3:**
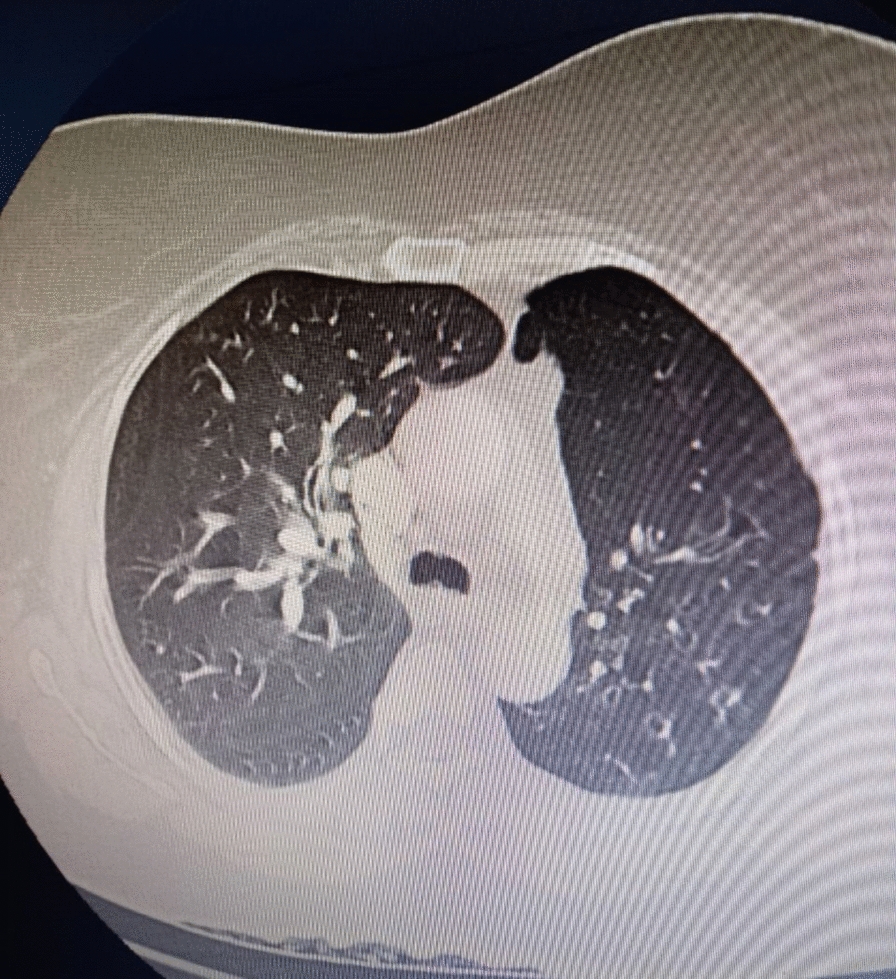
Chest computed tomography showed left lung hyperluceny and bronchiectasis

**Fig. 4:**
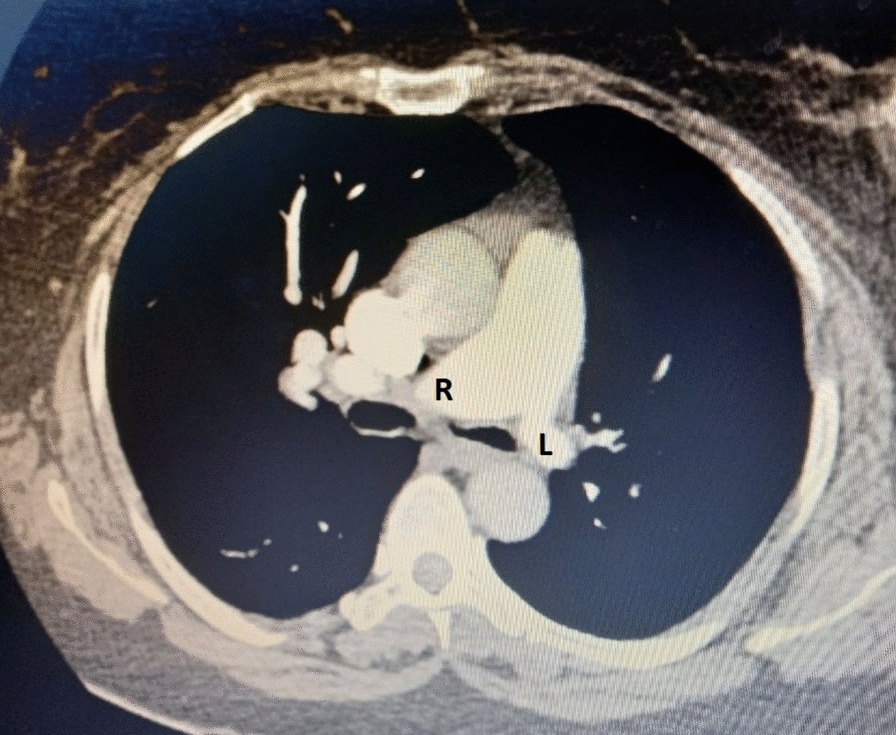
Chest angio-computed tomography (mediastinal window) displaying left pulmonary artery (L) hypoplasia

**Fig. 5:**
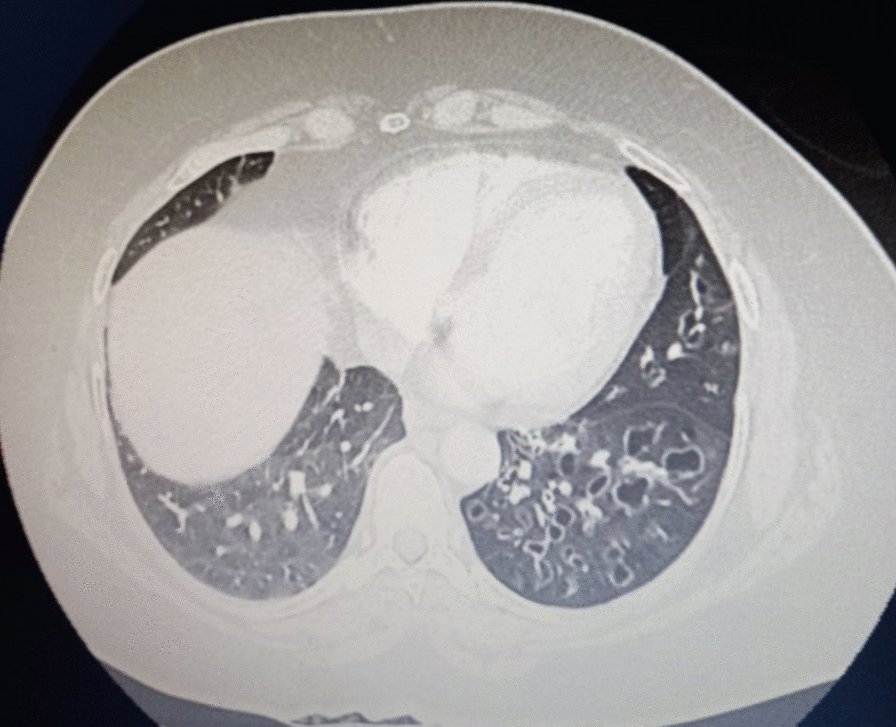
Chest computed tomography showing diffuse cystic bronchiectasis in the left lung

Chest computed tomography angiography (CTA) demonstrated left lung volume loss with hyperlucency (Fig. 3) and hypoplastic left pulmonary artery and veins (Fig. 4) and left lung diffuse varicoid and cystic bronchiectasis (Fig. 5) with superimposed bronchopneumonia. It was negative for pulmonary embolism, and echocardiogram was normal. These CTA findings were consistent with Swyer–James–Macleod syndrome. The patient started on intravenous antibiotics. She was advised about her condition and discharged with improvement.

When she was examined in the outpatient department a month after being discharged, her chest examination revealed scattered wheezing over the posterior chest, and she was experiencing intermittent productive coughing. Mucolytic, inhaled corticosteroids, and long-acting beta agonist were prescribed. She had symptomatic improvement and is now receiving regular outpatient care.

## Discussion

Swyer–James–Macleod syndrome is an extremely rare disorder usually characterized by unilateral hyperlucency. Although the exact etiology is unknown, it is now largely believed that it is a rare consequence of childhood respiratory infections, most commonly bronchiolitis obliterans or pneumonitis. A history of severe or recurring lower respiratory tract infections during infancy or childhood is present in more than half of SJMS patients. Generally, bronchiolitis obliterans impairs vascular development in an area, and obstruction and emphysema develop in small airways [6, 7, 11]. Bronchiolitis obliterans produces bronchial inflammation, fibrosis, and constriction, resulting in airway obstruction and hyperinflation. The degree of perfusion decreases as a compensatory mechanism in the area with restricted ventilation. When this occurs and persists during pulmonary development in infancy and childhood, it results in hypoplastic vascular regions as well as emphysematous, obstructive pulmonary areas [11].

SJMS patients and some cases of PE may exhibit unilateral hyperlucency [6, 7, 12]. Despite the fact that the diseases are vastly different, the comparable radiological appearances might be troublesome when PE is clinically suspected. In our case, the patient presented with symptoms and radiographic findings that initially suggested PE. Due to diminished vascularity and airway diameter, the afflicted lung looks hyperlucent on chest X-ray in SJMS. This hyperlucent look is caused by reduced perfusion and ventilation in the afflicted lung scan. PE is a potentially fatal illness brought on by blood clots obstructing the pulmonary arteries. Shortness of breath, coughing, and abrupt onset pleuritic chest pain are typical symptoms. PE is frequently diagnosed on the basis of clinical suspicion, D-dimer values, and imaging procedures such as chest CTA. A PE is accompanied with decreased vascularity in peripheral lung areas distal to the artery constricted by reflex vasoconstriction, as well as mechanical blockages caused by thrombi in the pulmonary artery and its branches. In other words, there may be unilateral hyperlucency or oligemia [13–15]. In our case, the use of CTA was essential in distinguishing SJMS from PE.

Differentiating SJMS from PE is important, as the management and prognosis of these conditions differ significantly. Distinguishing between pulmonary embolism and Swyer–James–Macleod syndrome can be confusing because they share similar symptoms, such as shortness of breath and chest pain. Swyer–James–Macleod syndrome is rare, so physicians may not be as familiar with it compared with PE. While PE typically presents with pulmonary artery filling defects on imaging, SJMS is characterized by unilateral hyperlucent lung fields with reduced vascularity. However, overlapping or atypical radiographic findings can occur, complicating the differentiation between the two conditions. In places with limited resources, using basic imaging tools can make it hard to make a clear diagnosis. Healthcare providers who do not have much experience with rare lung conditions such SJMS may misinterpret symptoms and X-ray results, leading to confusion with more common conditions such as PE.

Addressing these challenges through increased awareness, expanding access to advanced diagnostic resources, and seeking expert opinions can help improve the accurate differentiation between Swyer–James–Macleod syndrome and other lung conditions such as pulmonary embolism.

In our case, the initial presenting symptoms, ECG results, and chest X-ray findings, coupled with a lack of familiarity with Swyer–James–Macleod syndrome, posed challenges and led to confusion. Despite the absence of the classic Westermarck’s sign, the chest X-ray revealed hyperlucency in the left upper lung zone, initially misinterpreted as oligemia related to pulmonary embolism. The ECG findings, including T wave inversions in V1–V3 and sinus tachycardia, prompted consideration of submassive PE, adding another layer of complexity to the diagnostic process. Moreover, in our specific clinical setting, we did not have access to key biochemical studies such as brain natriuretic peptide (BNP), high-sensitivity cardiac troponin, and D-dimer. This constraint potentially influenced result interpretation and heightened the complexity of the diagnostic process. CTA performed at another facility has confirmed the findings of SJMS and has ruled out the presence of pulmonary embolism.

The Wells score indicated an intermediate risk level for our case. However, it was unable to distinguish between pulmonary embolism and Swyer–James–Macleod syndrome in this instance. While the Wells score can be a useful tool, it is not always accurate in identifying PE for several reasons. The performance of the Wells score may vary depending on the characteristics of the patient population being assessed, such as age, comorbidities, and pretest probability of PE. The score, as with any prediction rule, has limitations in terms of its sensitivity and specificity. It may not capture all cases of PE or may incorrectly classify patients as having or not having PE [16–18]. In summary, the presence of mimickers such as SJMS can complicate the diagnostic process for pulmonary embolism and may limit the accuracy of clinical prediction rules such as the Wells score. Healthcare providers should maintain a high index of suspicion for mimickers, conduct a comprehensive evaluation, and use clinical judgment to ensure accurate diagnosis and appropriate management in patients presenting with symptoms suggestive of PE.

In a review of the literature, Akgedik *et al*. have documented adult-onset SJMS presenting with symptoms and radiographic findings mimicking pulmonary embolism. They described six adult cases in which the patient presented with dyspnea. Their pulmonary radiographs revealed lung hyperlucency, and pulmonary embolism was initially suspected. On further investigation, the pulmonary artery and branches thereof exhibited parenchymal emphysema and hypoplasia, and thus SJMS was diagnosed [19]. In our case, the presentation was more acute with additional symptoms, including hemoptysis and chest pain.

Cases with SJMS are typically asymptomatic and are frequently identified incidentally when lung images are examined. When symptomatic, exertional dyspnea or recurrent lung infections are the most common presentations for patients. Wheezes, dyspnea on exertion, decreased exercise tolerance, cough with or without hemoptysis, and pleuritic chest pain are some of the other symptoms. Accompanying bronchiectasis is prevalent, and patients with saccular cystic bronchiectasis may experience recurrent lung infections. Severe clinical outcomes such as sepsis, spontaneous pneumothorax, acute lung infections, and pulmonary hypertension have also been linked to SJMS [6, 7, 11, 20, 21]. Our patient presented with complaints of sudden onset dyspnea, hemoptysis, and chest pain. A total of 4 months back she was treated for lung infection, and at current presentation she was found to have bronchiectasis.

The management is patient-centered and given through a multidisciplinary team approach. Management is the mainly conservative. Prevention, early identification, and treatment of recurrent respiratory infections are necessary. Mucolytics and chest physiotherapy are also important in patents with bronchiectasis. In addition, corticosteroids and inhaled bronchodilators are used. Surgical treatment may be necessary in selected cases [8, 21–23]. Our patient took antibiotics and received a mucolytic, inhaled corticosteroids, and bronchodilators.

## Conclusion

Adult-onset cases of SJMS are rare and can present with symptoms and radiographic findings mimicking pulmonary embolism. This case highlights the importance of considering SJMS as a differential diagnosis in adults presenting with suspected PE, particularly when typical risk factors are absent. A thorough medical history review and appropriate imaging studies are crucial for accurate diagnosis and appropriate management of these patients.

## Data Availability

The data that support this case report are available from the corresponding author upon reasonable request.

## Abbreviations

CTA: Computed tomography angiography
ECG: Electrocardiogram
PE: Pulmonary embolism
SJMS: Swyer–James–MacLeod syndrome

## References

[1] Ohri SK, Rutty G, Fountain SW. Acquired segmental emphysema: the enlarging spectrum of Swyer-James/Macleod’s syndrome. Ann Thorac Surg. 1993;56(1):120–4. 8328841 10.1016/0003-4975(93)90414-D

[2] Lucaya J, Gartner S, García-Peña P, Cobos N, Roca I, Liñan S. Spectrum of manifestations of Swyer-James-MacLeod syndrome. J Comput Assist Tomogr. 1998;22(4):592–7. 9676450 10.1097/00004728-199807000-00015

[3] Macleod W. Abnormal transradiancy of one lung. Thorax. 1954;9(2):147. 13179127 10.1136/thx.9.2.147 PMC1019360

[4] Swyer PR, James G. A case of unilateral pulmonary emphysema. Thorax. 1953;8(2):133. 13077508 10.1136/thx.8.2.133 PMC1019253

[5] Behrendt A, Lee Y. Swyer-James-Macleod syndrome. StatPearls: StatPearls Publishing; 2022. 32119329

[6] Sen HS, Taylan M, Abakay O, Sezgi C, Cetincakmak MG. Adult diagnosis of Swyer-James-Macleod syndrome: retrospective analysis of four cases. Respir Care. 2014;59(4):e51–4. 24026189 10.4187/respcare.02552

[7] Abba AA, Al-Mobeireek AF. Clinical spectrum of Swyer-James-Macleod syndrome in adults. Saudi Med J. 2003;24(2):195–8. 12682687

[8] Dirweesh A, Alvarez C, Khan M, Shah N. A unilateral hyperlucent lung-Swyer-James syndrome: a case report and literature review. Respir Med Case Rep. 2017;20:104–6. 28138424 10.1016/j.rmcr.2017.01.004 PMC5256677

[9] Khalil KF, Saeed W. Swyer-James-Macleod syndrome. J College Phys Surg Pakistan. 2008;18(3):190–2. 18460255

[10] Mehra S, Basnayake T, Falhammar H, Heraganahally S, Tripathi S. Swyer–James–MacLeod syndrome—a rare diagnosis presented through two adult patients. Respirol Case Rep. 2017;5(5): e00245. 28638618 10.1002/rcr2.245 PMC5473102

[11] da Silva PSL, Lopes R, Neto HM. Swyer-James-MacLeod syndrome in a surgically treated child: a case report and brief literature review. J Pediatr Surg. 2012;47(4):e17–22. 22498410 10.1016/j.jpedsurg.2011.12.011

[12] Coche E, Verschuren F, Hainaut P, Goncette L. Pulmonary embolism findings on chest radiographs and multislice spiral CT. Eur Radiol. 2004;14:1241–8. 14968257 10.1007/s00330-003-2203-2

[13] Westermark N. On the roentgen diagnosis of lung embolism. Acta Radiol. 1938;4:357–72.

[14] Algin O, Gökalp G, Topal U. Signs in chest imaging. Diagn Interv Radiol. 2011;17(1):18. 20669122 10.4261/1305-3825.DIR.2901-09.1

[15] Marshall GB, Farnquist BA, MacGregor JH, Burrowes PW. Signs in thoracic imaging. J Thorac Imaging. 2006;21(1):76–90. 16538167 10.1097/01.rti.0000189192.70442.7a

[16] Girardi AM, Bettiol RS, Garcia TS, Ribeiro GL, Rodrigues ÉM, Gazzana MB, *et al*. Wells and Geneva scores are not reliable predictors of pulmonary embolism in critically ill patients: a retrospective study. J Intensive Care Med. 2020;35(10):1112–7. 30556446 10.1177/0885066618816280

[17] Dresden S, Mitchell P, Rahimi L, Leo M, Rubin-Smith J, Bibi S, *et al*. Right ventricular dilatation on bedside echocardiography performed by emergency physicians aids in the diagnosis of pulmonary embolism. Ann Emerg Med. 2014;63(1):16–24. 24075286 10.1016/j.annemergmed.2013.08.016

[18] Klok FA, Mos IC, Kroft LJ, de Roos A, Huisman MV. Computed tomography pulmonary angiography as a single imaging test to rule out pulmonary embolism. Curr Opin Pulm Med. 2011;17(5):380–6. 21681098 10.1097/MCP.0b013e328348b3de

[19] Akgedik R, Karamanli H, Aytekin İ, Kurt AB, Öztürk H, Dağlı CE. Swyer–James–Macleod syndrome mimicking an acute pulmonary embolism: a report of six adult cases and a retrospective analysis. Clin Respir J. 2018;12(2):404–9. 27402385 10.1111/crj.12529

[20] Fregonese L, Girosi D, Battistini E, Fregonese B, Risso FM, Bava GL, *et al*. Clinical, physiologic, and roentgenographic changes after pneumonectomy in a boy with Macleod/Swyer-James syndrome and bronchiectasis. Pediatr Pulmonol. 2002;34(5):412–6. 12357493 10.1002/ppul.10178

[21] Koyama T, Osada H, Kitanaka Y, Funaki S, Hiekata T. Surgically treated Swyer-James syndrome. Jpn J Thorac Cardiovasc Surg. 2001;49:671–4. 11757341 10.1007/BF02912478

[22] Yekeler E. A rare case of swyer-james macleod syndrome and a new clinical presentation, acquired lobar emphysema. Ann Thorac Surg. 2012;93(5):e123–5. 22541234 10.1016/j.athoracsur.2011.11.050

[23] Daniel TL, Woodring JH, Mac Vandiviere H, Wilson HD. Swyer-James syndrome—unilateral hyperlucent lung syndrome: a case report and review. Clin Pediatr. 1984;23(7):393–7. 10.1177/000992288402300706 6723187

